# Supplementary figures and images for: Identification of Metabolite and Lipid Profiles in a Segregating Peach Population Associated with Mealiness in Prunus persica (L.) Batsch
Source: Metabolites. 2020 Apr 16;10(4):154. doi: 10.3390/metabo10040154 (PMC7240955; doi:10.3390/metabo10040154)

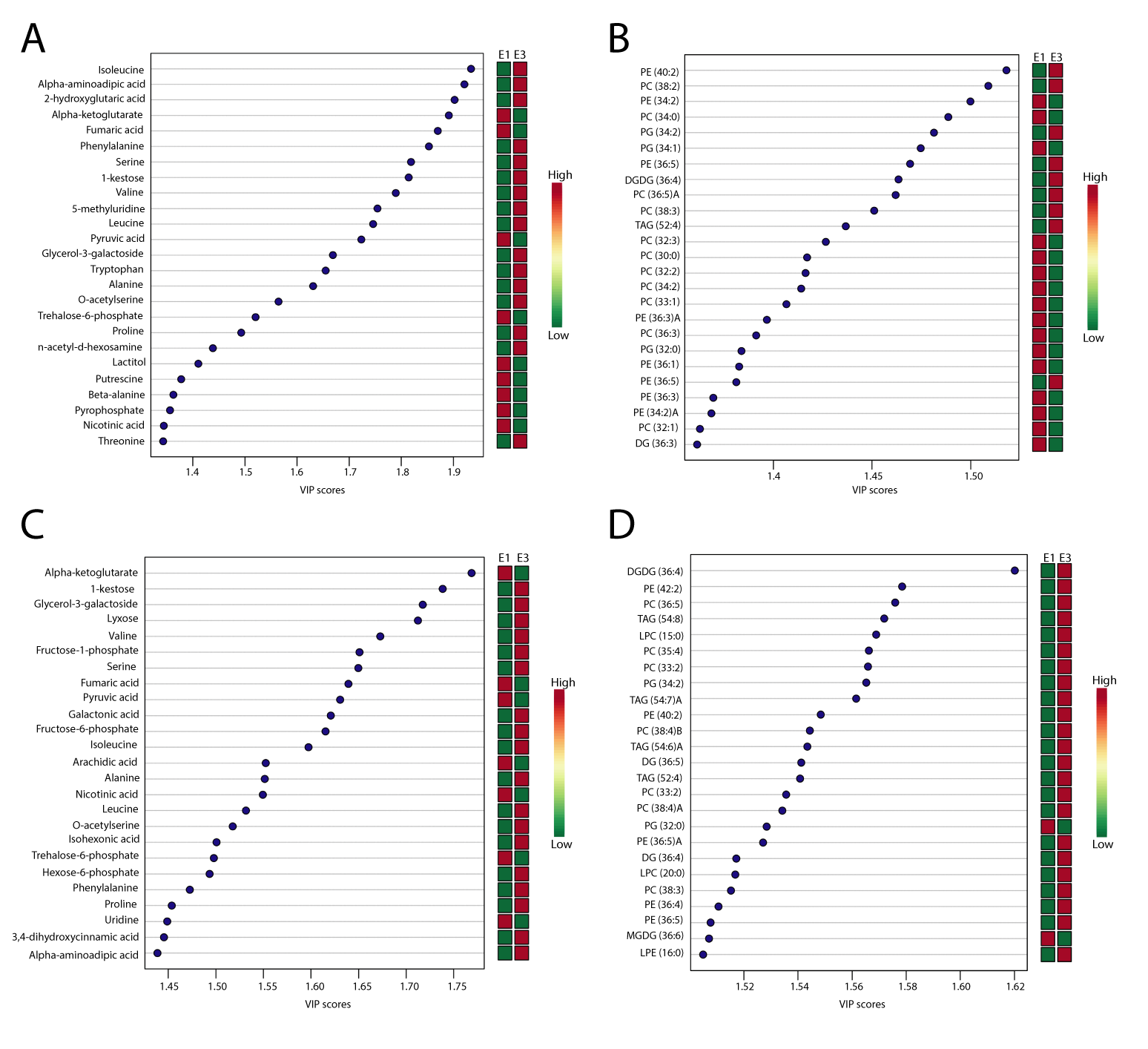

Supplement: Supplementary file 1 [file metabolites-10-00154-s001.zip › S3.tif]
